# Community RNA-Seq: multi-kingdom responses to living versus decaying roots in soil

**DOI:** 10.1038/s43705-021-00059-3

**Published:** 2021-12-06

**Authors:** Erin E. Nuccio, Nhu H. Nguyen, Ulisses Nunes da Rocha, Xavier Mayali, Jeremy Bougoure, Peter K. Weber, Eoin Brodie, Mary Firestone, Jennifer Pett-Ridge

**Affiliations:** 1 Physical and Life Sciences Directorate, Lawrence Livermore National Laboratory, Livermore, CA USA; 2 Department of Tropical Plant and Soil Sciences, University of Hawaiʻi at Mānoa, Honolulu, HI USA; 3 Department of Environmental Microbiology, Helmholtz Centre for Environmental Research, Leipzig, Germany; 4 Centre for Microscopy, Characterisation & Analysis, The University of Western Australia, Perth, Australia; 5 Earth and Environmental Sciences, Lawrence Berkeley National Laboratory, Berkeley, CA USA; 6 Department of Environmental Science, Policy and Management, University of California, Berkeley, CA USA; 7 Life and Environmental Sciences Department, University of California Merced, Merced, CA USA

**Keywords:** Microbial ecology, Microbial ecology

## Abstract

Roots are a primary source of organic carbon input in most soils. The consumption of living and detrital root inputs involves multi-trophic processes and multiple kingdoms of microbial life, but typical microbial ecology studies focus on only one or two major lineages. We used Illumina shotgun RNA sequencing to conduct PCR-independent SSU rRNA community analysis (“community RNA-Seq”) and simultaneously assess the bacteria, archaea, fungi, and microfauna surrounding both living and decomposing roots of the annual grass, *Avena fatua*. Plants were grown in ^13^CO_2_-labeled microcosms amended with ^15^N-root litter to identify the preferences of rhizosphere organisms for root exudates (^13^C) versus decaying root biomass (^15^N) using NanoSIMS microarray imaging (Chip-SIP). When litter was available, rhizosphere and bulk soil had significantly more Amoebozoa, which are potentially important yet often overlooked top-down drivers of detritusphere community dynamics and nutrient cycling. Bulk soil containing litter was depleted in Actinobacteria but had significantly more Bacteroidetes and Proteobacteria. While Actinobacteria were abundant in the rhizosphere, Chip-SIP showed Actinobacteria preferentially incorporated litter relative to root exudates, indicating this group’s more prominent role in detritus elemental cycling in the rhizosphere. Our results emphasize that decomposition is a multi-trophic process involving complex interactions, and our methodology can be used to track the trajectory of carbon through multi-kingdom soil food webs.

## Introduction

Soil carbon is derived primarily from root inputs, both living and detrital [1–4], and the fluxes that control the size of the soil carbon pool are critical to the global carbon (C) cycle. The soil adjacent to plant roots (the rhizosphere) is a nexus for root C input, microbial C transformation, as well as C loss through decomposition [5, 6]. Most root C is remineralized to CO_2_, and a substantial portion of the remainder undergoes microbial transformation before it has the opportunity to be stabilized via mineral associations [7]. The spatial organization of soil habitats such as the rhizosphere and detritusphere (regions surrounding decaying organic matter) is particularly important for carbon and nutrient exchanges amongst soil microbes, viruses, and fauna, and the characteristics and rates of these transformations determine how much carbon remains in soil [3]. While it is widely recognized that soil bacteria, fungi, and fauna are instrumental to decomposition [8], typically these groups are studied in isolation, and less is known about how the greater soil food web of bacteria, archaea, fungi, and microfauna responds to decomposing litter in the rhizosphere versus surrounding bulk soil.

To date, microbial ecology surveys studying litter decomposition that use amplicon sequencing have primarily focused on bacteria or fungi, but decomposition is conducted by a broad array of organisms [9] including microfauna (here we use this umbrella term to include protists, nematodes, and other soil invertebrates <100 µm) [8, 10]. It is widely understood that fungi play a key role in the decomposition of plant litter by providing the majority of the extracellular enzymes needed to depolymerize plant residues [11–15]. Litter-associated microfauna may consume and directly breakdown root litter [8], and protists and nematodes are also known to consume fungi and bacteria [10, 16–19]. Thus, the presence of these consumers can affect both soil microbial community composition and the rate of litter decomposition [20–26]. However, while interactions between roots and microbes have been intensively studied, we know little about the broader multi-trophic interactions among root-associated microbes and other members of the soil food web (fungi, fauna, and phage) that control the movement of C through soil [27]. To improve our predictive understanding of decomposition in soil, we need to characterize substrate preferences and trophic interactions amongst the broader soil food web.

In the past decade, amplicon metabarcoding with high-throughput sequencing approaches have allowed the identification of multiple groups of soil organisms [28–30]. However, PCR amplification has multiple layers of biases, including primer selection and bioinformatic processing, and the lack of universal primers means multiple primer sets are required to amplify taxonomically disparate groups [31–33]. An alternative approach is to use an amplification-independent method, such as shotgun RNA sequencing (RNA-Seq) for community analysis, which we call “community RNA-Seq”. This method not only reduces the inherent biases associated with PCR [34–36], but since rRNA is an integral part of ribosomes that controls protein synthesis across multiple domains of life [37], direct sequencing of RNA allows us to study active communities within Bacteria, Archaea, and Eukarya simultaneously without amplification. In addition, as most RNA is ribosomal RNA, the resulting sequences have naturally high coverage of the ribosomal subunits most frequently used for taxonomic analysis (e.g., 16S, 18S, 28S) [38], which allows a greater sequencing depth of taxonomic markers than metagenomic sequencing. Like other methods based on ribosomal marker genes, community RNA-Seq is affected by variation in ribosomal copy number; gene copies can substantially vary between organisms and cannot be used as measures of absolute abundance [39, 40]. To assess community composition, community RNA-Seq is followed by either reassembling rRNA fragments into full ribosomal subunits or directly classifying the reads [38, 41–43]. Community RNA-Seq has been used to a limited degree in microbial ecology due to the difficulty of extracting and working with soil RNA, but initial studies suggest that it is a particularly useful approach to study soil protists without PCR and cultivation biases [38, 44]. Eukaryotic primers are not universal for protists [45], which has led to Amoebozoa being underrepresented in SSU rRNA gene surveys (due to long SSU regions), and ciliates being overrepresented (due to short SSU regions) [44].

Methods that leverage isotopes as tracers of microbial activity (e.g. assimilation of substrates) are capable of adding another layer of ecological information to community surveys and can expand our understanding of food web dynamics and nutrient cycling in multi-trophic communities. Stable isotope probing (SIP) approaches are a powerful way to study microbial ecophysiology in complex environments [46, 47]. In a SIP study, a normally rare stable isotope (e.g., ^13^C, ^15^N, ^18^O) is added to an environmental sample and organisms that incorporate the labeled substrate become isotopically enriched in proportion to their activity [48, 49]. Nucleic acid-SIP techniques [48, 50] are currently the most widely used means to directly connect microbial identity to substrate utilization. An alternative to density-gradient SIP is ‘Chip-SIP’, where an imaging mass spectrometer (NanoSIMS) is used to determine the isotopic enrichment of RNA hybridized to a phylogenetic microarray [51, 52]. This method requires relatively low ^13^C enrichment (0.5 atom%) relative to density-gradient SIP, permits shorter isotope incubations, can assess both ^13^C and ^15^N enrichment in the same sample, targets RNA, and requires no amplification step.

In this study, we used community RNA-Seq (shotgun RNA sequencing) and Chip-SIP to study how living versus detrital root material affects “multi-kingdom” communities (colloquially defined as bacteria, archaea, fungi, and microfauna) in the *Avena fatua* rhizosphere and surrounding bulk soil. Using Chip-SIP, we traced the fate of ^13^CO_2_ after it was fixed by the plants and released as ^13^C-rhizosphere exudates, and simultaneously traced ^15^N-enriched decaying root litter (detritusphere) to determine the interactive effects of these two soil habitats and the substrate preferences of different taxa. We hypothesized that rhizosphere organisms that decompose litter would consume both litter and root exudates, rather than specialize on a single resource.

## Methods

### Microcosm setup and soil collection

**Fig. 1:**
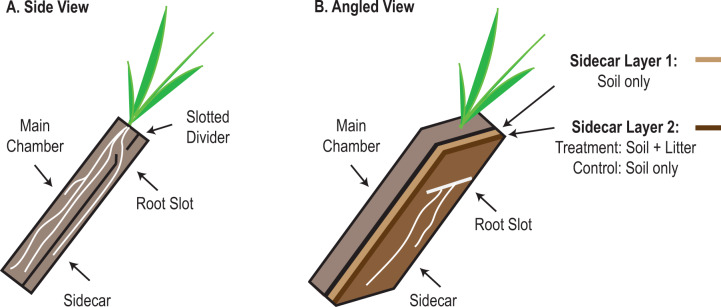
Microcosm design and sampling strategy. **A** Microcosms had a main chamber that housed the plant, *Avena fatua*, during plant growth and maturation. The main chamber was separated from an auxiliary root chamber (the sidecar) by a solid divider; microcosms were tilted to promote root growth along the outside face of the sidecar. After 6 weeks, the solid divider was removed and replaced with a slotted divider to permit root growth into the sidecar, and the sidecar was then filled with experimental soil. **B** Litter-containing microcosms (rhizosphere-litter, bulk-litter) were amended with ^15^N-labeled root detritus (Layer 2), which was placed on top of unamended soil (Layer 1). Unamended microcosms (rhizosphere-control, bulk-control) were prepared in the same manner, but no litter was added to Layer 2. After 6 days the roots entered the sidecar, and the plants were then pulse labeled for 3 days with ^13^CO_2_ and harvested. Rhizosphere soil (<2 mm from a root growing along the face of the sidecar) and bulk soil (>4 mm from root) were excised with a scalpel.

Soils were collected at the Hopland Research and Extension Center (HREC, GPS 38.992982, −123.067562) in Hopland, CA (USA), which experiences a Mediterranean climate [53] and exists on territory originally occupied by the indigenous Pomo Nation. Soils are a fine loam Alfisol complex (Ultic Haploxeralf mixed with a Mollic Palexeralf) with 1.7% C and 0.14% N [54]. The top 10 cm of soil was collected from beneath a stand of naturalized *Avena barbata* within a wild annual grassland community at 1 m intervals along a 10 m transect in January. Large plant material was removed, including root pieces, and soil was sieved to 2 mm, homogenized, then mixed with sand (1:1 w/w sand:dry weight soil) to improve drainage. The mixed soil was packed into the main chamber of 6 plastic microcosms (15 cm × 5 cm × 40 cm) to a density of 1.2 g/cm^3^ as previously described (Fig. 1A) [55, 56]. Briefly, two clear plexiglass rectangles were screwed onto a 5-cm-thick U-shaped frame; the screws allowed the outer panel to be removed for rhizosphere collection. A removable divider was placed into a slot within the interior of the frame; this divider separates the main chamber from the sidecar (5 mm deep, Fig. 1A). *A. fatua* seeds (Pacific Coast Seed Inc., Tracy, CA, USA) were germinated in the dark for 7 days. One seedling per microcosm was planted once the roots were greater than 1 cm long and after the shoot had emerged from the seed. Plants were grown in a greenhouse under a 14-h photoperiod and watered every 2–3 days to field water-holding capacity (approximately 50% saturation), which approximates spring conditions during a seasonably wet year. After 6 weeks, the solid divider separating the main chamber from the sidecar was replaced with a slotted divider (slots ca. 10 cm × 4 mm) and the sidecar was filled with the experimental soil (Fig. 1A).

Sidecar experimental soil was freshly collected and sieved HREC soil (not mixed with sand). Half of the microcosms received ^15^N-labeled *A. fatua* root litter chopped to ca. 1 mm (78 atom% ^15^N; see Supplemental Methods for details regarding production of this material). The ^15^N isotopic tracer allowed us to use mass spectroscopy to detect the communities that were actively consuming root litter-N. ^15^N-root litter was mixed into the soil by hand using clean nitrile gloves sterilized with ethanol. For the two litter treatments (rhizosphere-litter, bulk-litter; each with *n* = 3), the soil was added to the sidecar in two layers (each approximately 2.5 mm deep, Fig. 1B) to concentrate the litter in the same layer as the growing roots: Layer 1 contained 75 g of soil with no litter, while Layer 2 (rooting layer) contained 75 g of soil amended with 0.4 g of ^15^N-root litter. For the no litter treatments (rhizosphere-control, bulk-control; each with *n* = 3), Layer 2 did not include litter. After packing the sidecars, the microcosms were tilted at 45° with the sidecar facing down to encourage root growth into Layer 2 along the outside face of the sidecar.

After filling the sidecar, plants were grown for an additional 6 days prior to ^13^CO_2_ labeling, which is the amount of time it typically takes for roots to enter the sidecar. A 1.5 m × 1.5 m × 0.76 m plexiglass glovebox (Coy Laboratory Products, USA) was used as a labeling chamber at the UC Berkeley EPIC (Environmental Plant Isotope Chambers) facility [54]. The maximum chamber temperature was cycled between 26 and 28 °C during the day and allowed to cool naturally to 20 and 22 °C at night. Before dawn each day, the air in the chamber was cycled through a desiccator filled with soda lime to remove CO_2_ until the chamber atmosphere reached <25 ppm CO_2_. The chamber was then filled with 99 atom% ^13^CO_2_ until the concentration reached a set point of 400 ppm, and was maintained at 400 ppm throughout the day using an SBA-5 model IRGA (PP Systems, 400 ppm ^13^CO_2_ standard calibration) attached to a CR800 model datalogger (Campbell Scientific, Logan, UT, USA). Using this setup, the plants were labeled with ^13^CO_2_ for 3 days.

After 3 days of isotope labeling, the front plates of the sidecars were removed to access an intact rhizosphere along the entire length of a root. All rhizosphere soil within 2 mm of the roots was excised using a scalpel. The soils were immediately placed in ice-cold Lifeguard RNA protectant solution (MoBio, now Qiagen). Tubes were shaken for 2 min on a horizontal vortex adapter (MoBio, now Qiagen) on medium speed to release soil from the roots. The tubes were centrifuged at 2.5 × *g* for 1 min at 4 °C, and any roots or floating root litter were removed with flame-sterilized forceps. The remaining soil was pelleted by centrifuging at 2.5 × *g* for 5 min at 4 °C. After the supernatant was carefully removed, the pellets were immediately frozen on dry ice, and stored at −80 °C for molecular analysis. Soil >4 mm from a root was treated as bulk soil. To collect bulk soils with litter, the top half of the sidecars that contained ^15^N-labeled litter was randomly excised using a scalpel. These samples often contained visible pieces of ^15^N-labeled roots that were not removed from the collected sample. Bulk soil samples were processed in the same manner as rhizosphere soils. We collected a total of 12 soil samples: 2 locations (rhizosphere, bulk) × 2 litter conditions (litter, no litter) × 3 replicate microcosms. Hereafter, we refer to samples from the unamended control as “rhizosphere-control” and “bulk-control” and samples from the litter addition treatment as “rhizosphere-litter” and “bulk-litter”.

### RNA extraction and sequencing

For each sample, RNA was extracted in triplicate from 0.2 g soil using a phenol–chloroform extraction protocol [57], modified from Griffiths et al. [58]. Extracted nucleic acids were passed through the Allprep DNA/RNA Mini Kit (Qiagen Sciences, Maryland, USA) to separate RNA from DNA. RNA was treated with DNase using an on-column DNase digestion. For community RNA-Seq, metatranscriptomic libraries were prepared directly from total RNA without rRNA removal using the TruSeq RNA Kit (Illumina, Inc., San Diego, CA, USA) according to the manufacturer’s instructions. Metratranscriptomic libraries were sequenced on an Illumina GAIIX sequencer using 150 basepair (bp) paired-end sequencing at Lawrence Berkeley National Laboratory with an average of 9.5 million paired raw reads per sample. Sequences were deposited at NCBI under PRJNA692617.

### Sequence quality control and rRNA reconstruction

Sequences were demultiplexed, and sequence quality was checked with FastQC [59]. We used Trimmomatic [60] with default parameters with one exception; we removed the first 10 bp from the 5′ end due to overrepresentation of this region in the dataset. Sequences shorter than 60 bp after trimming were removed. Reads that did not pair were discarded. Code for sequencing processing and analysis is available at: https://github.com/enuccio/emirge_dataset.

EMIRGE [41] was used to reconstruct near-full-length SSU rRNA sequences for Bacteria, Archaea, and Eukarya using the script “emirge_amplicon.py”. The script was run on paired-end reads with the following parameters: mean insert length of 342, insert standard deviation of 100, and max read length of 151. The Greengenes 13_5 database clustered at 97% similarity was used to create the reference database for Bacteria and Archaea [61]. The SILVA 114 NR database [62] was used as a reference database for Eukarya. The database was also clustered at 97% as above. After the databases were created, the non-standard characters were altered as previously described [41]. Bowtie indices required by EMIRGE were calculated for the databases using bowtie-build [63]. Aligned sequences were trimmed to 1300 bp and converted to a format useable by UPARSE.

### OTU clustering and classification

Bacterial and archaeal sequences were analyzed separately from eukaryotic sequences. Sequences were clustered using UPARSE (usearch_v7) [64] and analyzed using QIIME 1.8 [65] at 97% sequence similarity. OTUs were classified using the RDP classifier [66], where bacterial and archaeal classifications were trained using Greengenes 13_5 and eukaryotic sequences were trained using SILVA 119NR [62]. UCHIME [67] was selected to detect chimeras after testing three chimera-checking tools (see Supplemental Methods). OTUs were required to be present in at least two samples, and OTUs classified as chimeras or plant and algal chloroplasts were removed from the dataset. In total, we analyzed 7229 unique full-length bacterial and archaeal RNA sequences created by EMIRGE (1127 OTUs at the 97% similarity level), and 8488 unique full-length eukaryotic RNA sequences created by EMIRGE (265 OTUs at 97% similarity level).

Since EMIRGE calculates a relative abundance estimate for each consensus sequence, a custom OTU table (sample × OTU matrix) was created after OTU picking to incorporate relative abundances of the consensus sequences into the microbial community analysis. To convert the consensus sequence relative abundance into sequence abundance, we multiplied the total number of reads that Bowtie mapped to the database by the relative abundance derived from the “normalized priors”, as per Miller et al. [68]: total mapped reads × consensus sequence relative abundance = number of sequences per consensus sequence. Since each OTU can contain multiple consensus sequences, we calculated the OTU sequence abundance by summing the number of sequences for each consensus sequence within the OTU. The samples were then rarefied to an even depth of 121 737 sequences for bacteria and archaea, and 27,668 sequences for eukaryotes. As per the recommendations of Miller et al. [68], OTUs with less than 0.01% relative abundance were removed.

### Statistical analysis

Community differences were visualized by non-metric multidimensional scaling (r package: metaMDS) using a pairwise weighted Unifrac distance matrix [69]. To determine which OTUs differed in relative abundance between the litter and unamended treatments, we performed two sets of parametric *t*-tests in QIIME (group_significance.py): rhizosphere-control vs. rhizosphere-litter; bulk-control vs. bulk-litter. Only OTUs that were detected in all three replicates of at least one treatment were considered for analysis. *P* values were corrected for multiple comparisons using a Benjamini–Hochberg correction. For simplicity, the grouping term “kingdom” was used to designate the level above phylum and to distinguish between fungal and protist groups. We note that while kingdom is still a commonly used and phylogenetically correct definition for Fungi [70, 71], the preferred terminology for protist taxonomy is supergroups followed by nameless ranks [72]. To calculate kingdom- or phylum-level relative abundances, relative abundances were summed for all OTUs within each group (kingdom for Eukarya, phyla for Bacteria and Archaea) and significant differences were determined using a *t*-test; in two instances where the data were non-normally distributed according to a Shapiro–Wilk test, we used a non-parametric Wilcoxon test (no significant differences found). Changes in the relative abundances for each group were determined by comparing litter-amended samples to their unamended control for bulk and rhizosphere soil separately (i.e., rhizosphere-control vs. rhizosphere-litter; bulk-control vs. bulk-litter).

### Chip-SIP analysis

To follow the assimilation of C and N from living plants and dead roots into the microbial community, we analyzed the rhizosphere of a microcosm containing both ^15^N-litter and ^13^C-exudates using Chip-SIP, a method that can detect and quantify ^15^N/^14^N and ^13^C/^12^C ratios of labeled RNA hybridized to a phylogenetic microarray [51, 52]. Detailed methods for probe design, microarray synthesis and hybridization, NanoSIMS analysis, and data processing can be found in Supplemental Methods. Briefly, we designed a microarray with probes using ARB [73] for the 180 most abundant Bacteria, Archaea, and Eukarya (fungi, protists, nematodes) OTUs found in this study, as well as probes targeting plant chloroplasts; a taxonomic summary of these probes is available in Table S1. Ten distinct probes per OTU were printed in three replicate blocks on the microarray. We produced two microarrays for this sample, one to detect RNA binding, and a second to detect RNA ^13^C and ^15^N isotopic enrichment with NanoSIMS high-resolution isotopic imaging. To detect RNA binding, RNA was labeled with Alexafluor 532 dye using the Ulysis kit (Invitrogen), fragmented with fragmentation buffer (Affymetrix), purified, concentrated, and hybridized onto the first array. This array, with fluorescently labeled RNA, was imaged with a Genepix 4000B fluorescence scanner. For the NanoSIMS analysis, unlabeled RNA was again fragmented, purified, and concentrated and then hybridized to a second array. This second array (with non-fluorescently labeled RNA) was also imaged with the fluorescence scanner to allow navigation to analysis spots in the NanoSIMS. Data were collected on the LLNL NanoSIMS 50 in pulse counting mode using aperture slit 3 and entrance slit 5, first collecting ^12^C^14^N^−^ and ^12^C^15^N^−^, and then ^12^C^14^N^−^ and ^13^C^14^N^−^. The resulting data were visualized as a stitched isotope map (Fig. S1) and data extracted as per Mayali et al. [51].

The proportion of isotopes is presented as a relative atom percent excess (APE) enrichment ratio of ^13^C to ^15^N (^13^C-APE:^15^N-APE) to indicate substrate preferences, where lower values indicate greater ^15^N enrichment in the RNA, and higher values indicate greater ^13^C enrichment in the RNA. Due to the higher background of ^13^C compared to ^15^N on the array, we used a normalization factor of 1.7 to calculate these relative enrichment ratios, as previously described [74]. Higher relative enrichment in ^15^N is interpreted as having a preference for amended ^15^N root litter, whereas higher relative enrichment in ^13^C is interpreted as having a preference for ^13^C root exudates. We emphasize that this ratio is a relative measure, as the ^13^C values do not reflect the total ^13^C ingested, since part of the ^13^C consumed is lost through respiration [74].

## Results

### Community structure from reconstructed SSU rRNA

**Fig. 2:**
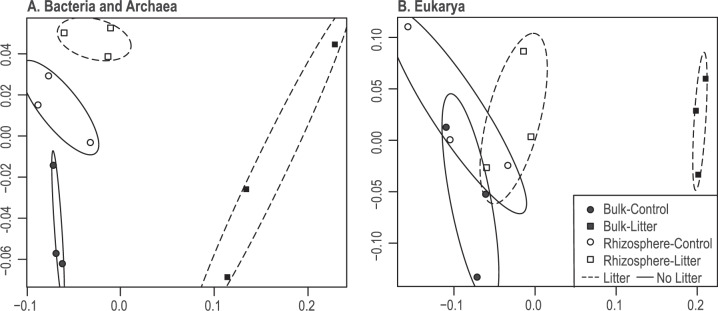
Multi-domain community structure by RNA-Seq. Community RNA-Seq non-metric multidimensional scaling ordinations are presented for **A** Bacteria and Archaea (assembled 16S rRNA), and **B** Eukarya (assembled 18S rRNA) in *Avena fatua* rhizosphere and bulk soil, in response to four growing root and root-litter amendment treatments (*n* = 3). Soil was sampled 3 days after fresh root growth into a microcosm auxiliary root chamber (sidecar). Filled symbols represent bulk soil, and hollow symbols represent rhizosphere soil. Squares indicate litter-amended soil treatments, and circles indicate soils with no added litter. Ovals represent the 95% standard error of the weighted average of scores per group (r package: ordiellipse) for litter (dashed lines) and no litter treatments (solid lines).

Both added root litter and living roots significantly altered the bacterial and eukaryotic community composition relative to bulk soil. Bacterial and eukaryotic communities had significantly different clusters per treatment by PERMANOVA analysis (Fig. 2) (see Table S2 for F Tables), though the eukaryotic communities had more overlap (Fig. 2B). The bulk-litter communities were the most distinct group for both bacteria and eukaryotes. Root litter had the strongest effect on both bacterial and eukaryotic communities, explaining 30% and 28% of the variance in community structure, respectively (two-way PERMANOVA: bacteria *F*_1,4_ = 7.2, *r*^2^ = 0.30, *p* > 0.001; eukaryotes *F*_1,4_ = 5.4, *r*^2^ = 0.28, *p* > 0.001). Living roots also significantly altered these communities; we measured a strongly significant rhizosphere effect for bacteria (two-way PERMANOVA: *F*_1,4_ = 4.7, *r*^2^ = 0.20, *p* = 0.006), and a slight but significant effect for eukaryotes (two-way PERMANOVA: *F*_1,4_ = 3.2, *r*^2^ = 0.17, *p* = 0.029).

### Phylum and kingdom level responses

**Fig. 3:**
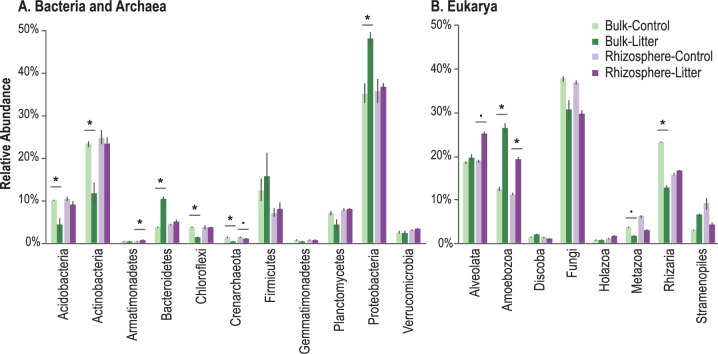
Relative abundance of bacteria, archaea, microfauna, and fungi in the rhizosphere and detritusphere. SSU rRNA relative abundance was aggregated at the **A** phyla level for Bacteria and Archaea, and **B** kingdom level for Eukarya in *Avena fatua* rhizosphere, bulk, and root-litter-amended soils. Relative abundance percentages were calculated relative to the total number of bacterial and archaeal sequences or eukaryl sequences, respectively. Treatments (*n* = 3) included: bulk soil with no litter amendment (bulk-control, light green), bulk soil amended with root litter (bulk-litter, dark green), rhizosphere soil with no litter amendment (rhizosphere-control, light purple), and rhizosphere soil amended with root litter (rhizosphere-litter, dark purple). Groups that significantly differed in relative abundance with litter amendments are indicated by * (*t*-test: *p* < 0.05) (bulk-control vs. bulk-litter; rhizosphere-control vs. rhizosphere-litter); “.” indicates marginal significance (*p* < 0.1).

In response to our soil treatments, we observed several significant changes in bacterial and eukaryotic relative abundance at the phylum and kingdom level, respectively. Proteobacteria and Actinobacteria had the highest relative abundance for bacteria in the rhizosphere (Fig. 3A). The relative abundances of Actinobacteria, Acidobacteria, and Chloroflexi were significantly reduced in the bulk-litter treatment (*t*-test: *p* < 0.05) (Fig. 3A), while the relative abundances of Bacteroides and Proteobacteria were significantly increased in the bulk-litter treatment. For the eukaryotes, Amoebozoa had a significantly higher relative abundance in the presence of litter in both rhizosphere-litter and bulk-litter soils compared to their respective unamended controls (Fig. 3B). In the bulk-control, the relative abundance of Rhizaria significantly increased. While the litter-containing treatments appear to have less Fungi, these differences were not significant (*p* > 0.5) compared to the bulk-control and rhizosphere-control treatments.

### Significant litter and rhizosphere responders

**Fig. 4:**
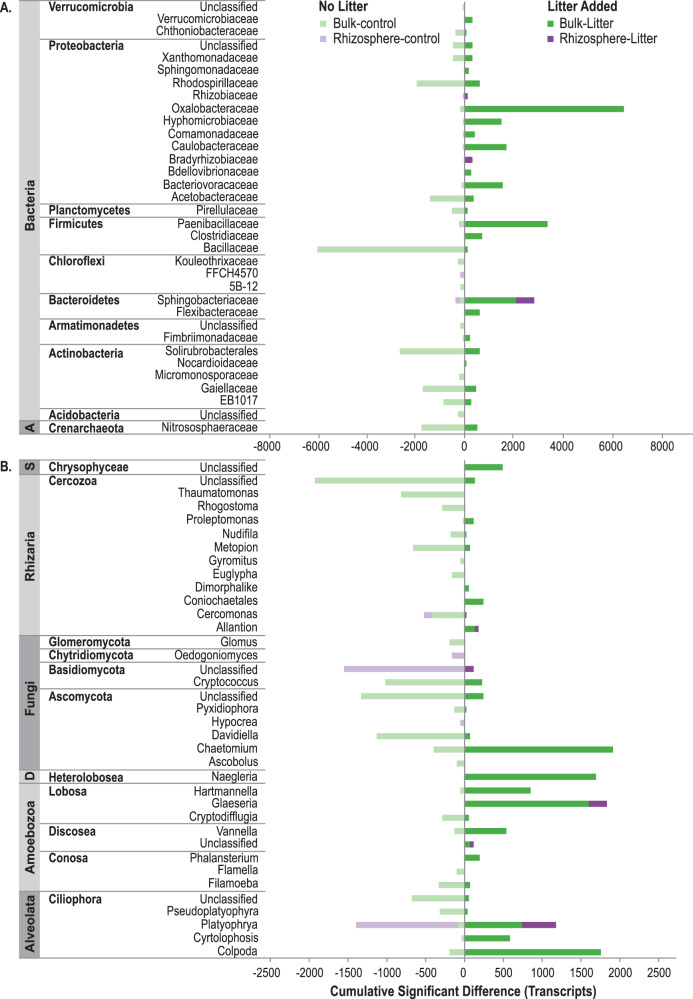
Cumulative significantly different transcripts for taxa that responded positively or negatively to root litter additions in bulk or rhizosphere soil. The effects of litter amendments were calculated separately for bulk soil and rhizosphere soil. Positive responses to litter are “dark green” for bulk soil and “dark purple” for rhizosphere soil. Negative responses to litter (or preference for unamended soil) are “light green” for bulk soil and “light purple” for rhizosphere soil. Transcripts were aggregated by **A **taxonomic family for Bacteria and Archaea and **B** genus for Eukarya; OTU transcript abundances were averaged across replicates (*n* = 3) prior to aggregation. Multiple comparisons were accounted for using a FDR *p* value correction. A = Archaea, S = Stramenopiles, D = Discoba.

To expose the unique effects of decaying roots on the soil microbiome, we compared litter-amended soil to the unamended controls for bulk soil and the rhizosphere. In bulk soil, litter additions significantly increased specific groups of protists, fungi, and bacteria, whereas litter amendments in the rhizosphere had fewer significant responders overall (Fig. 4). Protists from multiple lineages were more abundant in the presence of litter (Fig. 4B); *Colpoda* sp. (Alveolata), *Glaesaria* sp. (Amoebozoa), and *Naegleria* sp. (Heterolobosea) were some of the most abundant genera (Fig. 4B). Within the Amoebozoa, in addition to *Glaesaria* sp., *Hartmannella* sp. and *Vannella* sp. were also abundant in bulk-litter soil. *Platyophyra* sp. (Alveolata) were abundant in both rhizosphere-litter and bulk-litter soils. Of the Fungi, saprotrophic *Chaetomium* sp. (Ascomycota) responded the most strongly to litter, while other fungal taxa were more abundant in the absence of litter (*Cryptococcus* sp., *Davidiella* sp.). The bacterial taxa that strongly responded to the litter included *Massilia* sp. in the Oxalobacteriaceae (Proteobacteria), and OTUs in the families Paenibacilliaceae (Firmicutes) and Sphingobacteriaceae (Bacteroidetes) (Fig. 4A). When the rhizosphere was amended with litter, bacteria in the families Sphingobacteriaceae (Bacteroidetes), Bradyrhizobiaceae and Rhizobiaceae (alpha-Protoebacteria) significantly increased. Additional detailed taxonomic results can be found in Table S3.

When no litter was present, an unclassified fungus in the phylum Basidiomycota and *Platyophrya* sp. (Alveolata) responded strongly to the rhizosphere. Protists from the Rhizaria, (phylum Cercozoa) were more abundant in unamended soil, particularly unclassified genera within the classes Thicofilosea and Eugliphida. Of the Bacteria, taxa from the Rhodospirillaceae (Proteobacteria), Bacillaceae (Firmicutes), Solirubrobacterales (Actinobacteria) were most prominent in bulk-control soil.

### Chip-SIP: substrate preferences

**Fig. 5:**
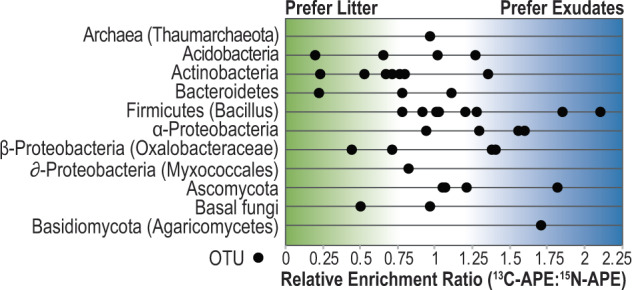
Relative substrate preferences for detrital ^15^N root litter versus ^13^C root exudates amongst bacterial, archaeal, and fungal populations detected by the Chip-SIP phylogenetic isotope array approach. Each dot represents an OTU that was significantly enriched in ^13^C or ^15^N derived from ^13^C-exudates or ^15^N-root detritus, and OTUs are organized in rows by phylum (or subclass for Proteobacteria). The *x*-axis is the ratio of the atom percent excess (APE) ^13^C enrichment and ^15^N enrichment for a set of ten phylogenetic probes (a unitless relative measure); the ratio was corrected by 1.7 to account for dilution of the C signal by the chip surface. The position of the taxon along the *x*-axis indicates its preference for exudates or root litter. Those that are positioned toward the left (green) incorporated relatively more isotope from ^15^N-litter whereas those positioned towards the right (blue) incorporated relatively more isotope from ^13^C-exudates. Probes targeting protists and nematodes were included on the chip (Table S1) but did not capture significantly enriched RNA after 3 days of *Avena fatua* ^13^CO_2_ labeling.

We used Chip-SIP stable isotope analysis to distinguish substrate preferences in bacterial, archaeal, and eukaryal taxa between root exudates (^13^C enriched) and decaying root litter (^15^N enriched). In the ^13^C-labeled rhizosphere amended with ^15^N-litter, we detected 42 OTUs with isotopically enriched RNA after 3 days of ^13^CO_2_ labeling (Fig. 5; 1 archaeon, 33 bacteria, 8 fungi) (Table S4); four probe sets for protists were included on the array, which detected low amounts of fluorescence but were not isotopically enriched (see probe taxonomy in Table S1). Of these four protist probe sets, two targeted a key group identified by our community RNA-Seq as a bulk-litter responder (Vannellida probes in the Discosea) and had low microarray fluorescence. The other two abundant protist responders (*Glaesaria*, *Hartmannella*) did not have Chip-SIP probe sets targeting these groups. Chip-SIP probe sequences, fluorescence values, NanoSIMS isotope ratio data, and representative fluorescence image are available in Table S5.

We did not detect any microbial RNA enriched solely in ^13^C or ^15^N, and only the plant-targeting probes on the array were solely enriched with ^13^C (they had the highest relative ^13^C/^15^N enrichment ratios in the dataset) (Table S4). As a phylum, the Actinobacteria OTUs contained a relatively higher proportion of ^15^N than ^13^C: 6 of 7 enriched taxa fell on the lower range of the ^13^C/^15^N spectrum (0.2–0.8) indicating that they were detritusphere organisms that preferred litter. Additional detritusphere organisms included Chitanophagaceae (Bacteroidetes) and 2 Oxalobacteraceae OTUs. Organisms that consumed both fresh and detrital plant material more equally during this 3-day study (relative enrichment ratios 0.8–1.2) included Thaumarchaeota, Dothideomycetes and Leotiomycetes fungi (Ascomycota), Xanthobacteraceae (alpha-Proteobacteria), and 3 *Bacillus* OTUs. Rhizosphere-dwelling organisms that appeared to prefer rhizosphere exudates (relative enrichment ratios 1.2–2.1) included two *Bacillus* OTUs, two Rhizobiales OTUs (Bradyrhizobiaceae, *Rhizobium*), two Burkholderiales OTUs (*Massilia*), Chaetothyriomycetidae fungi (Ascomycota), and Agaricomycetes (Basidiomycota).

## Discussion

While it is widely recognized that soil bacteria, fungi, and fauna are instrumental to organic matter decomposition [8], typically these groups are studied in isolation, and less is known about how the greater soil food web interacts with and is shaped by the availability of different organic substrates. To this end, we directly sequenced total RNA to identify bacteria, archaea, and eukaryotes in the presence and absence of root litter, and we determined how a living root altered these communities. We also used NanoSIMS-enabled microarray analysis (Chip-SIP) to track the fate of ^15^N-root litter and ^13^C root exudates and identified the substrate preferences of abundant organisms in a rhizosphere amended with litter.

### Substrate preferences in the rhizosphere versus detritusphere

In grassland systems, fresh root exudates and decaying roots exist in close proximity, which raises the prospect for cross-kingdom interactions and substrate niche differentiation [27, 57]. Our Chip-SIP results help to disentangle microbial substrate preferences in an active rhizosphere intersecting with an active detritusphere. We used two isotopic tracers to determine if soil microbes preferentially consumed ^13^C-exudates or ^15^N-litter. In support of our initial hypothesis, most rhizosphere organisms incorporated resources from both fresh root exudates and detrital inputs, but with different preferences for detrital or root inputs. Fungi tended to assimilate both ^15^N from litter and ^13^C-exudates equally, or even prefer ^13^C-exudates, supporting previous results that saprotrophic fungi can channel fresh rhizodeposits into the soil food web [75].

Interestingly, while Actinobacteria were the second-most abundant phylum in the rhizosphere treatments, Chip-SIP showed they tended to prefer ^15^N derived from litter and incorporated minimal ^13^C from rhizosphere exudates. This is consistent with recent findings in a similar system where Actinobacteria had the highest CAZyme gene expression in a relatively young detritusphere (<6 days old) and aging rhizosphere soils (>20 days old), but did not show significant gene expression in the young rhizosphere when ample root exudates were present (<6 days old) [57]. While density-gradient SIP studies show that Actinobacteria are active plant cellulose degraders [76–79], they may still benefit from metabolic handoffs in the rhizosphere during litter decomposition or N-mining, as auxotrophy was recently found to be more prevalent in Actinobacteria compared to other cellulolytic bacteria and crossfeeding might support growth [80]. Our results indicate that rRNA gene patterns alone have limited ability to infer substrate preferences and additional information is necessary to assess microbial ecophysiology, such as through isotope tracing or activity-based analyses.

### Rhizosphere and bulk soil harbor unique litter-decomposing communities

Environmental microbiology has long been consumed by the question of what maintains the enormous phylogenetic and functional diversity in complex ecosystems such as soil. In our previous work, we identified that bacterial rhizosphere and detritusphere communities form distinct guilds defined by CAZy gene expression over time in the *Avena* rhizosphere, where coexistence in soil was facilitated by niche differentiation based on substrate preferences [57]. Similarly, in this study, each treatment was dominated by a unique microbial and microfaunal community, with community assembly driven by habitat and substrate preferences. Substrate-based guilds defined using ^13^C^15^N-Chip-SIP agreed with gene-based guilds defined using CAZy gene expression [57] for the *Massilia* (Rhizosphere guild), which preferred ^13^C-exudates, and also for the Bacteroidetes and Actinobacteria (Detritusphere guild), which preferred ^15^N-litter. Interestingly, we identified detritusphere populations in the rhizosphere that appeared to incorporate isotopes from litter and root exudates relatively equally; this group included members of the Thaumarchaeota, Xanthobacteraceae, *Bacillus* species, and Dothideomycetes and Leotiomycetes fungi. The closest relative to the Thaumarchaeota in our dataset is *Nitrosocosmicus* (Silva accession FJ784305); a strain of this genus was previously shown to be a ureolytic soil archaeal ammonia oxidizer that can grow on organic urea alone [81]. In our 3-day study, it was unclear whether these organisms were accessing both ^13^C-exudates and ^15^N-litter through urea consumption or through uptake of inorganic mineralization byproducts (^13^CO_2_, ^15^NH_3_). As we only analyzed one timepoint, these patterns of substrate-based niche differentiation may further change and diversify with time and plant growth [27, 54, 82–84].

### Amoebae—a potentially important yet overlooked top-down driver of detritusphere community dynamics

Our community RNA-Seq analysis found that protists were abundant in decomposing litter in both bulk soil and the rhizosphere, and Amoebozoa and Alveolata had the highest relative abundance in our detritusphere soils. A previous metatranscriptomics study found that Rhizaria and Amoebozoa were abundant in grassland soils relative to peatlands [44]. Interestingly, we observed that Rhizaria were abundant predominantly in unamended bulk-control soil, whereas Amoebozoa were more abundant when litter was present in both bulk soil and the rhizosphere. Our results suggest that these two abundant groups may occupy different niches within the soil environment. Previous work has also shown that protist micropredators, including Amoebozoa, can have pronounced niche differentiation in rhizosphere and bulk soil [85].

Microfaunal predation is generally overlooked as a top-down driver of microbial community assembly [86]. Amoebae, in particular, are known to be mycophagous or bacterivores [17, 18] and can influence microbial community structure [13, 87]. In our study, of the Amoebozoa groups that responded significantly to the bulk-litter treatment, *Hartmannella* sp. and *Vannella* sp. are reported as exclusive bacterivores [88], while *Glaesaria* sp. are omnivores that can consume some amount of root litter [88, 89], though they can also be grown on bacteria alone [89]. In a complementary transcriptomics dataset from this soil [57], *Acanthamoeba* expression of exoproteases (enzymes that degrade extracellular protein) was highest in the litter-containing rhizosphere and bulk soils (Fig. S2); these organisms are also reported to be omnivores [88] and further suggests that omnivorous protists play an active role in detritusphere microbial community dynamics by consuming litter, microbial biomass, or both. Since the Amoebozoa Supergroup is typically missed in amplicon analyses [90], our results suggest Amoebae may be overlooked contributors to detritusphere microbial community dynamics and nutrient cycling.

### Relevance of the microbial food web for soil C cycling

Multi-trophic communities are critical to the breakdown of plant-derived organic matter, and the community changes we observed may have altered the flows of carbon in soil. Microbial communities have diverse physiological strategies for gaining access to and assimilating carbon substrates [9]. In our study, niche differentiation of the soil bacteria, fungi, and microfauna may have altered the rates of decomposition and the composition of the resulting degradation products [9, 91], including: altering soil exoenzyme composition, the types of organic matter degraded, and the resulting breakdown products in each treatment [92, 93]. For example, we observed that Actinobacteria decreased in the presence of litter while *Chaetomium* fungi increased; this likely altered the composition of exoenzymes available to breakdown plant material, as well as the diversity of compounds available for further microbial processing or sorption to mineral surfaces [94].

Selective predation by protists or Bacteriovoraceae can also significantly alter both the taxonomic and the functional composition of the soil microbiome [86, 95], but it is currently unclear how micropredation impacts the flows of soil carbon. In soil, decomposition is thought to be propelled by bacteria, fungi, mesofauna (e.g., microinvertebrates), and macrofauna (e.g., earthworms, millipedes) whose shredding action creates smaller particles that are more readily accessible to microbes [8]. On the other hand, many soil microfauna (e.g. protists, nematodes) primarily consume bacteria and fungi or are omnivores [19, 96–98], and can directly or indirectly impact C flows by assisting with litter decomposition, altering the populations of available decomposers, or altering nutrient cycling. In our study, it is possible that the reductions of fungi and Actinobacteria in bulk-litter soil (both potential decomposers) might have been driven in part by micropredator grazing [99, 100]. Micropredators can accelerate the turnover of microbial biomass and also excrete nutrients derived from microphagy [13, 101], which can indirectly enhance litter decomposition [26]. Protists can stimulate microbial nutrient cycling through the so-called ‘microbial loop’ [102, 103], a phenomenon where N contained in microbial biomass is higher than the N demand of protists, and predation ultimately leads to an increase in available N after excretion. Previous work has also shown that micropredation may diversify and alter forms of carbon available in soil; protists can selectively retain particular classes of metabolites during the digestion of microbial polymers [104]. Determining how these multi-kingdom interactions impact the flows and persistence of carbon in soil is a key goal for future work.

### Evaluation of methods used in this study

We found that community RNA-seq is a useful way to conduct a multi-kingdom community analysis in soil and believe this method could be made even more powerful by leveraging new assembly methods or improved sequencing technologies. Our EMIRGE ribosomal assembles tended to yield full-length sequences that we typically classified down to the family level. Recently, methods in addition to EMIRGE [41, 68] have been developed to assemble ribosomal proteins, such as REAGO [105], RAMBL [106], MATAM [107], and most recently MetaRib [42]. Alternatively, assembly could be skipped by using long-read sequencing, which could potentially yield results with even finer taxonomic resolution. For example, PacBio long-read sequencing can now provide full-length 16S sequences with subspecies resolution [108].

Chip-SIP is a unique method that can identify the ecophysiology of organisms and is compatible with the simultaneous use of multiple tracers, but as with all microarray-based methods, it can only target organisms with matching probes on the microarray. The rigors of NanoSIMS analysis necessarily limit the number of probes that can be screened, which is typically limited to approximately 200 organisms (2000 probes) in our experience [51]. Though we strove to include a broad representation of abundant microbial and macrofaunal lineages on our microarray, we did not include probes for some of the dynamic Amoebozoal groups. Future work could use detailed community RNA-Seq results to design a targeted probe set for Chip-SIP analysis.

## Conclusions

Using ‘community RNA-Seq’ metatranscriptomic sequencing of total RNA, we identified organisms in the three domains of life that responded to detrital root litter in rhizosphere and bulk soils. Litter-decomposing communities differed depending on the presence and absence of a growing root, and litter-amended bulk soil had the most distinct microbial and protist communities. Litter-amended rhizosphere and bulk soils contained significantly more Amoebozoa than unamended soil, highlighting that grazing by protists may be an important top-down control in detrital microbial communities, and that micropredator grazing should be considered when designing future bacterial and fungal litter decomposition studies. Chip-SIP NanoSIMS analysis showed that some abundant rhizosphere taxa preferentially used resources from nearby decaying root litter, which is an insight that could not have been discerned from compositional analyses alone. Future work combining shotgun RNA community analyses and stable isotope tracing can improve our ability to track nutrients and carbon through specific populations in multi-trophic food webs.

## Supplementary information

Supplementary Information

Table S1

Table S2

Table S3

Table S4

Table S5
