# Supplementary material for: Community RNA-Seq: multi-kingdom responses to living versus decaying roots in soil
Source: ISME Commun. 2021 Dec 6;1:72. doi: 10.1038/s43705-021-00059-3 (PMC9723751; doi:10.1038/s43705-021-00059-3)
Supplement: Supplementary file 1 — Supplementary Information [file 43705_2021_59_MOESM1_ESM.docx]

**SUPPLEMENTAL INFORMATION** *Nuccio et al. 2021 ISME Comms*

**METHODS**

**Production of ^15^N-labeled root litter**

To create ^15^N-labeled root litter, *Avena fatua* was grown for 8 weeks in fritted clay and fertilized solely with a custom mixture of Hoagland’s plant nutrient solution, where the nitrogen-containing compounds were replaced with their 99 atom % ^15^N analogs (0.505 g/L K^15^NO_3_ (Cambridge Isotope Laboratories), 0.59 g/L Ca(^15^NO_3_)_2_ x 4H_2_O (Cambridge Isotope Laboratories), 0.0225 g/L Sprint 330 iron chelate (Becker Underwood), 0.493 g/L MgSO_4_ x 7H_2_O, 0.080 g/L ^15^NH_4_^15^NO_3_ (Cambridge Isotope Laboratories), 2.86 g/L H_3_BO_3_, 1.81 g/L MnCl_2_ x 4H_2_O, 0.22 g/L ZnSO_4_ x 7H_2_O, 0.051 g/L CuSO_4_, 0.09 g/L H_3_MoO_4_ x H_2_O, and 0.5 ml/L of 1M KH_2_PO_4_ (pH 6.0)). Roots were triple washed in deionized water, dried, and stored for 1 year prior to use. Roots were chopped to ca. 1 mm lengths using scissors.

**Data processing using EMIRGE**

Since EMIRGE probabilistically reconstructs sequences, the near full-length sequences have a higher error rate than sequences directly generated by Illumina sequencing. To address this, singletons and sequences present in only one sample were removed from the dataset. Next, we assessed the efficacy of three chimera checking tools to identify potential chimeras created by EMIRGE (UCHIME (1), DECIPHER (2), Chimera Slayer (3)), and determined that UCHIME was the most effective chimera-checking tool for our dataset. To assess the chimera-checking tools, we used a set of EMIRGE sequences reconstructed from a mock community composed of 52 known isolates available as a supplemental dataset from Miller, Baker (4). Any novel sequences in this EMIRGE dataset are likely chimeras, since the SSU sequences of all the community members are known. We determined that 3 of 23 reconstructed sequences were putative chimeras, since they were < 90% similar to organisms in the NCBI database using BLAST. We tested each chimera-detection method to determine if it could identify these 3 sequences in the EMIRGE dataset. Only UCHIME was capable of detecting chimeras in the dataset; it was able to identify 2 of the 3 putative chimeras. Therefore, UCHIME was used for chimera-checking analyses.

**Probe design:** We designed a custom Chip-SIP array for rhizosphere soil with probes targeting bacteria, archaea, and eukaryotes (fungi, protists, nematodes). Probes were designed from the sequences reconstructed by EMIRGE from the four treatments in this experiment. The 180 most abundant OTU from bacteria, archaea, and eukarya were targeted for probe design. The microarray probes were designed in ARB using the following restrictions: (≤ 2 mismatches tolerated, GC content < 80%, homopolymer runs ≤ 4bp) (5). Twenty-five different probes were selected for each sequence that were unique relative to the SILVA database and RNA-Seq databases. Based on preliminary fluorescence data (using soils from actual experiment), from these 25 probes, we selected the 10 probes that had the highest hybridization scores to synthesize on the final microarray (signal:noise ≥ 1.3). Sequences that had few probes with positive fluorescence (signal:noise < 1.3) were added to the microarray by keeping 10 probes with the best ARB score (a measure of how specific the probe is to the sequence of interest).

**Chip-SIP microarray synthesis:** Microarrays were coated with a conductive surface prior to probe synthesis to eliminate charging during SIMS analysis. Glass slides coated with indium-tin oxide (Sigma) were treated with an alkyl phosphonate hydroxy-linker to provide a starting substrate for probe synthesis (6). Microarray probes (spot size = 17 μm) were synthesized using a photolabile deprotection strategy (7) on a Nimblegen Maskless Array Synthesizer (Roche). The probe sets were laid out in horizontal lines across the chip. All the probes were printed three times in three replicate blocks on the microarray. Nimblegen synthesis reagents (Roche) were delivered through the Expedite system (PerSeptive Biosystems).

**Microarray hybridization:** Two microarrays are necessary for Chip-SIP: a standard fluorescence microarray and a separate NanoSIMS microarray. Fluorescence analysis is necessary to confirm that the probes are hybridized with RNA. However, labeling RNA with a fluorophore introduces ^12^C-carbon that dilutes the ^13^C signal. Therefore, the RNA samples were split for fluorescence and NanoSIMS analyses and the RNA used in the NanoSIMS analysis was left unlabeled. Microarrays were not replicated.

For fluorescence analysis, the RNA was labeled with Alexafluor 532 dye using the Ulysis kit (Invitrogen), and were incubated for 10 min at 90°C (2 μL RNA, 10 μL labeling buffer, 2 μL Alexafluor reagent) and subsequently fragmented. RNA for NanoSIMS analysis was not labeled, and instead preceded directly to fragmentation. Samples were fragmented using 5X fragmentation buffer (Affymetrix) for 10 min at 90°C. Fragmented RNA was purified using a Spin-OUT™ minicolumn (Millipore), and RNA was concentrated by ethanol precipitation to a final concentration of 500 ng μL^-1^. For array hybridization, RNA samples were mixed with 1X Hybridization buffer (Nimblegen) and placed in a Nimblegen X4 mixer slide. The arrays were incubated inside a Maui hybridization system (BioMicro Systems) for 18 hrs at 42°C and then washed according to manufacturer’s instructions (Nimblegen).

Arrays with fluorescently labeled RNA were imaged with a Genepix 4000B fluorescence scanner at pmt = 650 units. Arrays with non-fluorescently labeled RNA were marked with a diamond pen and also imaged with the fluorescence scanner to subsequently navigate to the analysis spots in the NanoSIMS. Slides were trimmed and mounted in custom-built stainless-steel holders.

**Chip-SIP NanoSIMS analyses:** Mass resolution was set to ~10,000 mass resolving power to minimize the contribution of isobaric interferences to the species of interest (e.g., so that ^11^B^16^O^-^ contributes < 1/100 of the ^13^C^14^N^-^ ratio, and ^13^C_2_^-^ contributes < 1/1000 of the ^12^C^14^N^-^ ratio). Analyses were performed in imaging mode to generate digital ion images of the microarray for each ion species. The primary beam current was 5 to 7 pA Cs^+^, which yielded a spatial

resolution of 200-400 nm and a maximum count rate on the detectors of ~300,000 cps ^12^C^14^N. Analysis area was 50 x 50 µm^2^ with a pixel density of 256 x 256 with 0.5 or 1 ms/pixel dwell time. Ion counts were corrected for detector dead time on a pixel-by-pixel basis.

**Chip-SIP statistical analyses:** Individual 50 x 50 µm^2^ isotope ratio images were stitched together to create an isotope map of the microarray surface using custom software developed for NanoSIMS analysis (L’image, L. Nittler, Carnegie Institution of Washington). Probe spot regions of interest (ROIs) were selected by hand or with an autodefinition function, and ^15^N/^14^N and ^13^C/^12^C isotope ratios were calculated for each ROI. Isotope ratios were converted to atom percent excess (APE) values using the formula APE = [R_meas_/(1 + R_meas_) – R_control_/(1 + R_control_) ] × 100%, where R_meas_ is the isotope ratio measured by NanoSIMS and R_control_ is the mean ^15^N APE or ^13^C APE value for the control probe locations. The presence of RNA on each probe was confirmed using a separate fluorescence microarray analysis, where hybridized probes had a signal to noise ratio > 1.3.

Two criteria identified which OTUs were enriched with ^13^C or ^15^N. First, each probe set was required to have five of ten probes with a signal:noise ratio > 1.3; this determined which OTUs were present in the dataset. Second, each probe set had to have five probes with enrichment of either >0.020 ^13^C atom percent excess (APE) or >0.011 ^15^N APE (equivalent to 30‰ for both). For the OTUs that met these criteria, we calculated a ^13^C-APE:^15^N-APE ratio for the entire probe set by averaging all APE enrichments > 0 for ^13^C and ^15^N and then dividing the two averages (average ^13^C APE / average ^15^N APE).

A. ^15^N APE B. ^13^C APE


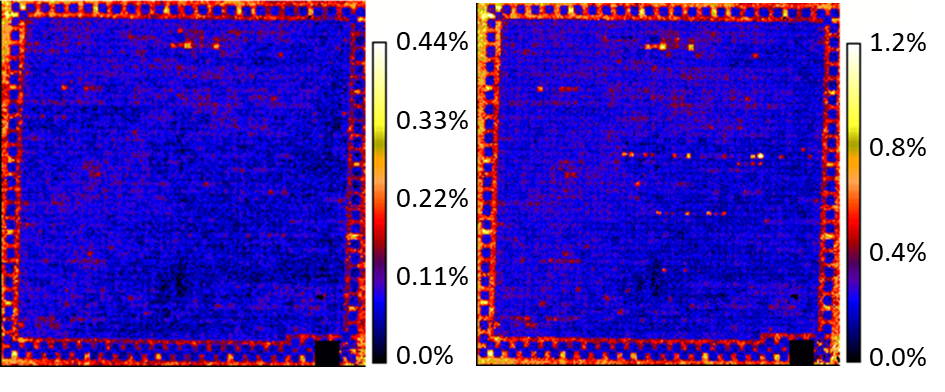


**Figure S1.** Chip-SIP isotope maps of a single phylogenetic microarray hybridized with isotopically labeled RNA from a rhizosphere soil microbial community exposed to ^15^N-labeled root detritus and ^13^C-exudates. As the microbes consume the substrates with the different isotope labels, they assimilate the isotopes into their microbial biomass and nucleic acids, and their preference for the ^15^N-root litter or ^13^C-exudates is determined by the amount of (A) ^15^N and (B) ^13^C contained in the RNA hybridized to a probe set specific to each taxon. Color scale bars indicate atom percent excess (APE) enrichment of the microarray surface. Probes sets are arranged in horizontal lines on the chip. The brightest ^13^C-enriched probe sets with no visible corresponding ^15^N-enriched probes are from plant host RNA.


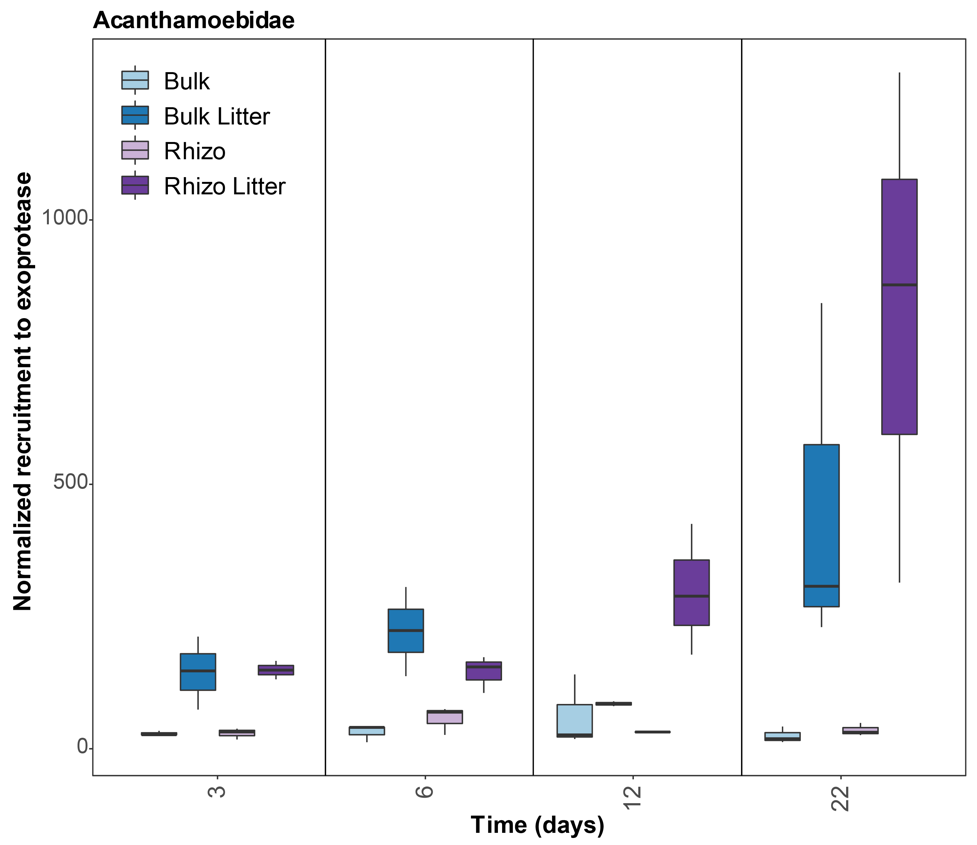


**Figure S2.** Acanthamoebidae exoprotease gene expression from a complimentary transcriptomic dataset on the same soil (8) from 3-22 days. Rhizosphere and bulk soil were amended with detrital root litter (Rhizo Litter, Bulk Litter) or unamended (Rhizo, Bulk). Sequences were normalized using DESeq2 as previously (8).

**REFERENCES**

1. Edgar RC, Haas BJ, Clemente JC, Quince C, Knight R. UCHIME improves sensitivity and speed of chimera detection. *Bioinformatics* 2011; **27**: 2194-200.

2. Wright ES, Yilmaz LS, Noguera DR. DECIPHER, a search-based approach to chimera identification for 16S rRNA sequences. *Appl Environ Microbiol* 2012; **78**: 717-25.

3. Haas BJ, Gevers D, Earl AM, Feldgarden M, Ward DV, Giannoukos G, et al. Chimeric 16S rRNA sequence formation and detection in Sanger and 454-pyrosequenced PCR amplicons. *Genome Res* 2011; **21**: 494-504.

4. Miller CS, Baker BJ, Thomas BC, Singer SW, Banfield JF. EMIRGE: reconstruction of full-length ribosomal genes from microbial community short read sequencing data. *Genome biology* 2011; **12**: R44.

5. Ludwig W, Strunk O, Westram R, Richter L, Meier H, Yadhukumar, et al. ARB: a software environment for sequence data. *Nucleic Acids Res* 2004; **32**: 1363-71.

6. Pett-Ridge J, Hoeprich P, Weber P, Brodie E, inventors; Lawrence Livermore National Security, assignee. Devices, Methods and Systems for Target Detection. USA2011.

7. Singh-Gasson S, Green RD, Yue Y, Nelson C, Blattner F, Sussman MR, et al. Maskless fabrication of light-directed oligonucleotide microarrays using a digital micromirror array. *Nat Biotechnol* 1999; **17**: 974-8.

8. Nuccio EE, Starr E, Karaoz U, Brodie EL, Zhou J, Tringe SG, et al. Niche differentiation is spatially and temporally regulated in the rhizosphere. *ISME J* 2020; **269**: 1-16.
